# Antioxidant and anti-inflammatory activities of durian (*Durio zibethinus* Murr.) pulp, seed and peel flour

**DOI:** 10.7717/peerj.12933

**Published:** 2022-02-07

**Authors:** Narin Charoenphun, Wiyada Kwanhian Klangbud

**Affiliations:** 1 Faculty of Science and Arts, Burapha University Chanthaburi Campus, Thamai, Chanthaburi, Thailand; 2 School of Allied Health Sciences, Walailak University, Thasala, Nakhon Si Thammarat, Thailand; 3 Center of Excellence research for Melioidosis and Microorganisms, Walailak University, Thasala, Nakhon Si Thammarat, Thailand

**Keywords:** Durian, Monthong, Chanee, Antioxidant, Anti-inflammation

## Abstract

The unripe pulp, inner peel and seed of durian were used in this study. These are generally not considered edible and must be disposed of as waste. However, they are good sources of bioactive compounds. Flour extracts from the unripe pulp, inner peel, and seed of two durian (*Durio zibethinus* Murr.) varieties, namely, Monthong and Chanee, were analyzed chemically to determine their total phenolic content (TPC), antioxidant, and anti-inflammatory capacities. Chanee pulp (CPu) contained a higher TPC (5285.37 ± 517.65 mg GAE/g) than Monthong pulp (MPu), Monthong peel (MP), Monthong seed (MS), Chanee peel (CP) and Chanee seed (CS) (*p* = 0.0027, 0.0042, 0.0229, 0.0069 and 0.36), respectively. The antioxidant activity of each durian extract was determined against ABTS, nitric oxide, superoxide, hydroxyl, and metal ions. The results indicated that the pulp, inner peel and seed of these durian varieties had antioxidant capacities. Murine Raw 264.7 macrophages were used to determine the cytotoxicity of the flour extracts. The extract of CS flour had the lowest cytotoxicity followed by MP, CPu, CP, MPu and MS (*p* = 0.5926, 0.44, 0.3191, 0.1471 and 0.0014), respectively. The anti-inflammatory activity was tested by anti-nitric oxide (NO) production in lipopolysaccharide (LPS) stimulated cells by co-treating the Raw 264.7 cells with each durian flour extract and LPS. The extract of MP flour had the lowest IC_50_ against NO production, indicating the highest anti-NO production activity followed by CS, CPu, MPu, CP and MS (*p* = 0.7473, 0.0104, < 0.0001, 0.0002 and < 0.0001, respectively). The information obtained in this study is useful for researchers to explore more durian varieties in Southeast Asia to find bioactive compounds that might be novel nutraceuticals for antioxidant, anti-inflammation and therapeutic functional food.

## Introduction

Durian (*Durio zibethinus* Murr.) is a member of the *Bombaceae* family, and it is one of the well-known tropical fruits in Southeast Asian countries, including Thailand, Malaysia, and Indonesia. In Thailand, durian is often regarded as the king of fruits (Booncherm & Siriphanich, 1991; Suwanagul, 1990). However, while more than 20 durian varieties are under cultivation, only a few important varieties, including *Durio zibethinus* Murr., cv. Monthong and Chanee are popular among durian growers and consumers (Pensiri & Visutsak, 2017). The shapes of durian fruits are either round or oval, while their peel is thick. The peel, especially the outer part, is semiwoody and completely covered with very strong and sharp thorns (Hokputsa et al., 2004). The inner peel is edible, but it is rarely eaten.

A durian fruit generally has approximately 3–5 seeds surrounded by edible custard-like pulp (also called flesh or aril). The durian pulp is the primary part of the fruit that is consumed. The pulp has an approximately 10–35% pulp-to-seed ratio (Husin et al., 2018). A sulfur odor, sweet flavor and yellowish color are the specific characteristics of durian pulp (Hokputsa et al., 2004). Previous studies reported that durian pulp is an excellent source of various nutrients. According to Devalaraja and colleagues (2011), durian pulp contains 1.47% protein, 5.33% fat, 3.1% fiber and 27% carbohydrate (Devalaraja, Jain & Yadav, 2011). Durian pulp is also rich in vitamins and minerals, including vitamin A, vitamin C, vitamin B6, folic acid, thiamin, riboflavin, niacin, calcium, potassium, iron, sodium, magnesium, phosphorus, and zinc (Ho & Bhat, 2015). Moreover, its pulp contains a good balance of essential fatty acids, including 35.93% stearic acid, 32.91% palmitic acid, 9.50% palmitoleic acid, 4.86% 10-octadecenoic acid, 4.68% oleic acid, 2.52% myristic acid, and 2.20% linoleic acid (Ho & Bhat, 2015).

Because only ripe fruits are consumed, the harvest of unripe fruits, both intentionally and unintentionally by farmers, is very problematic and can damage the durian industry. Furthermore, unripe fruits are not sellable, and if they reach consumers, they are not accepted because they are not tasty. According to Booncherm & Siriphanich (1991), the percentage of unripe fruits in the durian market in Thailand could be higher than 30%. The present study made flour from the unripe pulp of two durian varieties (Monthong and Chanee). In previous studies, immature durian pulp has been used in food processing in other forms, such as durian cookies and chips to produce snacks with high nutrition. Moreover, important substances can be extracted from unripe durian pulp, seeds and peels and used as food ingredients (Charoenphun & Kwanhian, 2019). The processing of unripe durian is a value-adding approach to the fruits during harvest and can be used to create a variety of food.

Durian seeds and inner peels (inner shells) are considered to be waste after removing the edible parts. Previous studies have reported the nutritional and health benefits of durian seeds and inner peels (Ho & Bhat, 2015). However, the white endosperm of Durian seeds is covered with a brown seed coat. Therefore, it is usually discarded despite its usefulness. Mat Amin and colleagues reported making flour from durian seed, which can be used as an ingredient in food products such as cake and cookies. It has a high fiber content and nutrition (Mat Amin & Arshad, 2009). In addition, durian seed flour can also be used as a thickening agent due to the presence of hydrocolloids and starch. Moreover, durian seed was found to have only 1.8% fat. It was reported to contain 12.2% palmitic acid as its major fatty acid, followed by 8.42% oleic and 6.50% linoleic acids (Berry, 1980).

Durian peel has been previously reported to have an excellent nutritional composition and contain many bioactive compounds. Feng and colleagues reported that durian peel had triterpenoids and glycosides with anti-inflammatory activity that could inhibit lipopolysaccharide-induced NO production in the RAW 264.7 cell line (Feng et al., 2018). The extract from the Monthong cultivar had greater antioxidant and anti-inflammatory activities than that prepared from the Chanee cultivar (Chingsuwanrote et al., 2016). Durian peel has antioxidant effects, which may be related to the flavonoids and phenolic compounds contained in the durian peel. The coumarin compound propacin in durian peel can significantly inhibit the release of NO and prostaglandin E2 (PGE2) induced by LPS in RAW264.7 cells (Zhan et al., 2021).

Information on the health benefits of flour produced from the peel, pulp and seed of these durian varieties is not available. This information is important for utilizing unripe durian to make flour that can be used in health-promoting products. Therefore, the present study aimed to investigate the antioxidants and anti-inflammatory activities of extracts from durian flour made from immature pulp, peel, and seeds of the major Thai durian cultivars, Monthong and Chanee. The results of the present study will be able to solve the problem of unripe durian and reduce agricultural waste during durian production by transforming the peel and seeds into value-added products. Durian flour could be used as a food ingredient or functional food beneficial to health.

## Materials and Methods

### Durian flour preparation and extraction

Two durian (*Durio zibethinus* Murr.) varieties, Monthong and Chanee, were selected for this study. The time required to ripen durians fully depends on the durian variety. The ripening date is predicted from the day after flowering and is used to determine the optimal harvest time. The maturation periods of Monthong and Chanee are 140 days and 120 days, respectively. Durian fruits were purchased from a local market in Chanthaburi Province, Thailand. There were 20-30 fruits of each variety. The unripe fruits were separated into pulp, inner peel and seed. The unripe (immature) pulp, inner peel and seed were cut into small pieces and then oven-dried for 18 h at 60 °C or until the moisture was lower than 12% by weight. Then, the dried fruits were ground by a grinder (Dxfill, Model DXM-500, China) at a speed of 2 for 2 min. The flour samples were passed through sieves of 100 mesh. Six flour types, Monthong pulp (MPu), Monthong peel (MP), Monthong seed (MS), Chanee pulp (CPu), Chanee peel (CP) and Chanee seed (CS), were stored in vacuum plastic bags until analysis, which was undertaken a few days after drying.

The flour samples were extracted in 95% ethanol (10 grams of flour per 100 mL of ethanol) for 1 h. The extracts were filtered by Whatman™ Grade 4 Filter Paper (Merck KGaA, Darmstadt, Germany). The extraction procedure for each sample was performed three times until 300 mL of ethanol in total had been added. The extracts were dried by a vacuum evaporator, and the dry samples were weighed. The flour extracts were then dissolved in 95% ethanol for the antioxidant activity study.

### Determination of total phenolic compounds

The flour extracts were analyzed for total phenolic compounds according to the method described by Singleton & Rossi (1965). Briefly, 2.5 mL of 10% Folin-Ciocalteu reagent was added to 0.5 mL of the extract. After the sample and reagent were mixed, 2.0 mL of 4% sodium carbonate was added, mixed well and incubated at room temperature for 2 h. The absorbance was measured at 740 nm. The result was used to calculate the equivalent mg gallic acid/g of the extract (Singleton & Rossi, 1965).

In this study, a standard gallic acid with a known value was used. The standard gallic acid was diluted at different concentrations, and it was tested using the same method as that applied to the durian extracts. The absorbance values of gallic acid were plotted against the concentrations of gallic acid in a linear equation in which the *Y*-axis was the absorbance and the *X*-axis was the concentration. The *Y*-axis was replaced by the absorbance values of the extracts to obtain the concentrations of the extracts. Then, the calculated concentration from the linear equation was used to calculate the total phenolic content (mg GAE/g) as follows:

Total phenolic content = (C × V)/m,

where C is the concentration obtained from the standard curve of gallic acid, V is the volume of the extract used in the assay and m is the mass of the extract in g.

### Determination of antioxidant activities

The antioxidant activity of each durian extract against radicals, including ABTS, nitric oxide, superoxide, hydroxyl and metal ions, was measured. The SC_50_ (50% radical scavenging concentration) was calculated from the linear equation between the percentage of % radical inhibition and the durian extract concentration.

The ABTS (2,2-azinobis(3-ethylbenzothiozoline)-6-sulfornic acid) scavenging activity was determined based on a method described previously (Re et al., 1999). ABTS^+^ radicals are generated when ABTS and potassium persulfate react together for 12–16 h at room temperature. The ABTS^+^ radical was diluted with ethanol until the absorbance at 734 nm was 0.7 ± 0.02. One milliliter of diluted ABTS^+^ radical was mixed with 10 µL of durian flour extract or standard Trolox. The reaction occurred for 30 min at 30 °C. The absorbance at 734 nm was measured, and the reducing absorbance was remeasured after 3 min to calculate ΔA/min. The % ABTS radical scavenging was calculated as:

[(A_control_−A_test_)/A_control_] ×100,

where A_control_ is ΔA/min of the control reaction and A_test_ is ΔA/min in the presence of the durian extract.

The nitric oxide scavenging assay was undertaken according to a previously described method (Natarajan et al., 2009). The Griess reagent was used to test nitric oxide (NO) synthesized by sodium nitroprusside (SNP) (1% sulfanilamide, 0.1% naphthylethylenediamine dichloride and 3% phosphoric acid). SNP in aqueous solution at physiological pH spontaneously generates NO, which interacts with oxygen to generate nitrite ions that can be estimated using a Griess reagent. Scavengers of NO compete with oxygen, leading to reduced production of NO. The extract was combined with 10 mM SNP in phosphate-buffered saline (PBS) and incubated at 25 °C for 3 h. The solution mixtures were reacted with Griess reagent. The absorbance was measured at 540 nm and referenced to the absorbance of ascorbic acid, which was used as a positive control. The % nitric oxide scavenging was calculated as:

[(A_control_−A_test_)/A_control_] ×100,

where A_control_ is the absorbance of the control reaction and A_test_ is the absorbance in the presence of the extracts.

The superoxide radical scavenging assay was measured by the reduction of nitro blue tetrazolium (NBT) according to a previously reported method (Fontana, Mosca & Rosei, 2001). The nonenzymatic phenazine methosulfate-nicotinamide adenine dinucleotide (PMS/NADH) system generated superoxide radicals, which reduce NBT to purple formazan. One milliliter mixture contained phosphate buffer (20 mM, pH 7.4), NADH (73 µM), NBT (50 µM), PMS (15 µM) and various concentrations of extracts. After incubation for 5 min at room temperature, the absorbance was measured at 560 nm against a blank to determine the quantity of the generated formazan. All tests were replicated three times. The % superoxide radical scavenging was calculated as:

[(A_control_−A_test_)/A_control_] ×100,

where A_control_ is the absorbance of the control reaction and A_test_ is the absorbance in the presence of durian extracts.

The hydroxyl radical scavenging assay was performed following the method of Halliwell and colleagues (Halliwell, Gutteridge & Aruoma, 1987) with some modifications. The extract was mixed with Haber–Weiss reaction buffer (1 mM EDTA pH 7.4, 10 mM FeCl_3_, 10 mM H_2_O_2_ and 1 mM L-ascorbic acid) and 10 mM deoxyribose in a total volume of 1.0 mL. The mixture was incubated at 37 °C for 1 h. After that, it was heated at 80 °C for 30 min, and one mL of 2-TBA (0.5% 2-TBA in 0.025 M NaOH, 0.02% BHA) and one mL of 10% trichloroacetic acid (TCA) were added and it was incubated for another 45 min at 95 °C. The absorbance of the mixture was measured at 532 nm after cooling. The % hydroxyl radical scavenging was calculated as:

[(A_control_−A_test_)/A_control_] ×100,

where A_control_ is the absorbance of the control reaction and A_test_ is the absorbance when durian extracts are present.

The metal ion chelating assay was estimated according to the method suggested by Dinis and colleagues (Dinis, Maderia & Almeida, 1994) with some modifications. Briefly, 0.1 mL of 2 mM FeCl_2_ was added to 0.8 mL of different concentrations of the extract. The reaction was initiated by the addition of 0.1 mL of 5 mM ferrozine solution. The reaction mixture was vigorously shaken and then left at room temperature for 10 min. The absorbance was measured at 562 nm. The percent inhibition of the ferrozine-Fe2^+^ complex formation was calculated as:

[(A_0_−A_s_)/A_s_] ×100,

where A_0_ is the absorbance of the control and A_s_ is the absorbance of the extract/standard.

### Cell culture

The murine macrophage RAW 264.7 cell line (ATCC CRL-2278), provided by Dr. Jitbanjong Tangpong, was cultured in complete Dulbecco’s modified Eagle medium (DMEM) supplemented with 10% fetal bovine serum (FBS), 100 U/mL penicillin and 100 µg/mL streptomycin at 37 °C in 5% CO_2_. Cells were cultured in 96-well plates (2  × 10^4^ cells/well) for 24 h before treatment.

### Cell viability

Cell viability was assessed using a modified 3-(4,5-dimethylthiazol-2yl)-2,5-diphenyl-2H-tetrazolium bromide (MTT) assay (Mosmann, 1983). Briefly, cells (2  × 10^4^ cells/well) were seeded in a 96-well plate and treated with durian flour extract (1.56, 3.125, 12, 5, 25, 50, 100 and 200 µg/mL) for 48 h. Then, 100 µL of 5 mg/mL MTT (Sigma–Aldrich, St. Louis, USA) solution in phosphate-buffered saline was added to each well and further incubated for 4 h at 37 °C. Subsequently, 100 µL of dimethyl sulfoxide was added to each well to dissolve any deposited formazan. The absorption at a wavelength of 540 nm was measured with a microplate reader (Bio-Rad Laboratories Inc., Hercules, CA, USA). The CC_50_ (50% cytotoxicity concentration) was calculated from the linear equation between the percentage of cell viability and the extract concentration.

### Quantification of nitric oxide production

The nitric oxide (NO) concentration in the culture medium was determined using a Griess reaction kit (Sigma–Aldrich, St. Louis, MO, USA). Briefly, 2  ×10^4^ Raw 264.7 macrophage cells/well were cultured for 24 h. Then, the cells were cotreated with 100 µg/mL lipopolysaccharide (Sigma–Aldrich, St. Louis, USA) and durian flour extract (1.56, 3.125, 6.25, 12.5, 25, 50 and 200 µg/mL) for 24 h, and 100 µl supernatant was collected from each well. It was mixed with 100 µl Griess reagent (Sigma–Aldrich, St. Louis, USA) in new 96-well plates. After the mixture was incubated for 15 min at room temperature, the optical density was determined at 550 nm using a microplate reader (Bio-Rad Laboratories, Inc., Hercules, CA, USA). The NO concentration was calculated based on the standard curve of NO. The IC_50_ (50% inhibition concentration) of NO production was calculated from the linear equation between the NO concentration and the extract concentration.

All experiments were conducted in triplicate. The mean ± standard deviation (SD) was calculated for all parameters.

### Statistical analysis

The significant differences between the means for total phenolic content, antioxidant activities, and anti-nitric oxide production were determined by independent *t-tests*. A *p value* less than 0.05 was considered significant. GraphPad Prism version 8.0.1 was used for statistical analysis and graph creation.

## Results

### Total phenolic content of the durian flour extracts

**Figure 1:**
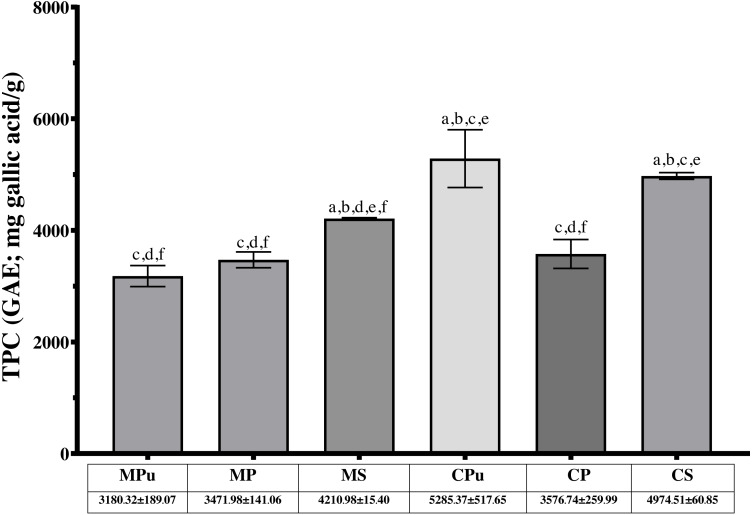
Total phenolic contents (TPC) of six durian flour extracts. ^a^ Significantly different from MPu, ^b^ significantly different from MP, ^c^ significantly different from MS, ^d^ significantly different from CPu, ^e^ significantly different from CP, and ^f^ significantly different from CS.

The highest total phenolic content (TPC) (5285.37 ± 517.65 mg GAE/g) was recorded in flour extract from the pulp of Chanee (CPu), whereas the lowest TPC (3180.32 ± 189.07 mg GAE/g) was found in the flour extract from the pulp of Monthong (MPu). Among the flour extracts of Monthong, Monthong seed (MS) had a significantly higher TPC (4210.98 ± 15.40 mg GAE/g) than Monthong peel (MP) and Monthong pulp (MPu), with *p* = 0.0007 and 0.0008, respectively. For the Chanee flour extracts, Chanee pulp (CPu) had the highest TPC, followed by Chanee seed (CS) and Chanee peel (CP), with *p* = 0.3600 and 0.0069, respectively. When TPC values in the same parts of the durian fruits were compared, flour extract from CPu had a significantly higher TPC than MPu (*p* = 0.0027). Flour extract from CS also had a significantly higher TPC than MS (*p* < 0.0001). Flour extracts from the peel of the two durian varieties were not significantly different for TPC (*p* = 0.5728). Moreover, flour extracts from all parts of the Chanee fruits had higher TPCs than those of the Monthong fruits. Figure 1 shows the level of TPC in the 6 Durian flour extracts. The *p values* are in the raw data Supplementary File.

### Antioxidant activity of durian flour extracts

**Figure 2:**
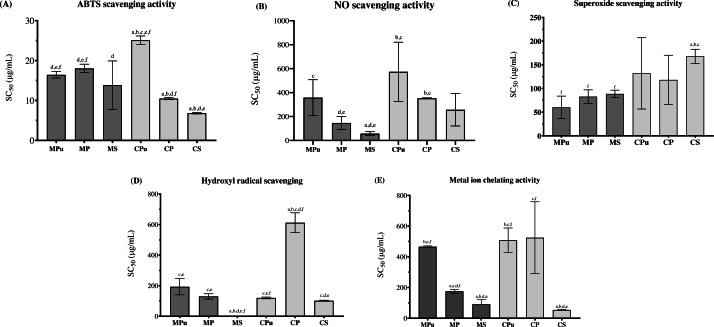
The antioxidant activities of durian flour extracts represent by the SC_50_(50% oxidant scavenging concentration) which determined by ABTS radical scavenging activity (A), nitric oxide scavenging activity (B), superoxide scavenging activity (C), hydroxyl radical scavenging activity (D) and metal ion chelating activity (E). ^a^ Significantly different from MPu, ^b^ significantly different from MP, ^c^ significantly different from MS, ^d^ significantly different from CPu, ^e^ significantly different from CP, and ^f^ significantly different from CS.

Flour extract from Chanee seed (CS) had the highest antioxidant activity as determined by ABTS radical scavenging activity relative to MPu, MP, MS, CPu and CP, with *p* < 0.0001, <0.0001, 0.115, <0.0001, and <0.0001, respectively. The SC_50_ values of ABTS radical scavenging activity of MPu, MP, MS, CPu, CP and CS were 16.43 ± 0.81, 18.03 ± 1.06, 13.85 ± 6.06, 25.11 ± 1.05, 10.48 ± 0.26 and 6.83 ± 0.19 µg/mL, respectively (Fig. 2A). The highest antioxidant activity determined by nitric oxide (NO) scavenging activity was recorded in the flour extract from MS, followed by those from MP, CS, CP, MPu, and CPu, with SC_50_ values of 57.08 ± 16.31, 145.80 ± 54.13, 256.30 ± 135.40, 351.90 ± 3.76, 357.70 ± 149.60 and 573.30 ± 247.30 µg/mL, respectively (Fig. 2B). The *p values* are 0.0824, 0.0646, <0.0001, 0.0258 and 0.0266, respectively. The highest antioxidant activity determined by superoxide radical scavenging activity was observed in the flour extract of MPu, followed by those of MP, MS, CP, CPu and CS, with SC_50_ values of 60.25 ± 23.74, 82.73 ± 14.36, 88.55 ± 7.97, 118.10 ± 52.13, 132.0 ± 75.28 and 167.90 ± 14.95 µg/mL, respectively (Fig. 2C). However, most of the results were not significantly different from MPu, with *p* = 0.2331, 0.1219, 0.1551, 0.1905 and 0.0027, respectively.

Flour extract from MS had significantly higher antioxidant activity determined by hydroxyl radical scavenging activity than MPu, MP, CPu, CP and CS with *p* = 0.0034, 0.0002, <0.0001, <0.0001 and <0.0001, respectively. The SC_50_ values of superoxide radical scavenging activity were 2.28 ± 0.22, 193.40 ± 53.25, 129.90 ± 17.67, 120.10 ± 5.46, 612.50 ± 64.59 and 101.90 ± 3.50 µg/mL, respectively (Fig. 2D). Flour extract from CS had the most significantly higher antioxidant activity determined by metal ion chelating activity than MS, MP, MPu, CPu, and CP, with SC_50_ values of 53.60 ± 2.70, 90.77 ± 30.08, 175.40 ± 12.55, 466.0 ± 4.91, 508.60 ± 79.12 and 524.60 ± 234.0 µg/mL, respectively (Fig. 2E). The *p values* are 0.1000, <0.0001, <0.0001, 0.0006 and 0.0252, respectively. The full set of *p values* is in the raw data supplementary file.

### Cytotoxicity of durian flour extracts

**Figure 3:**
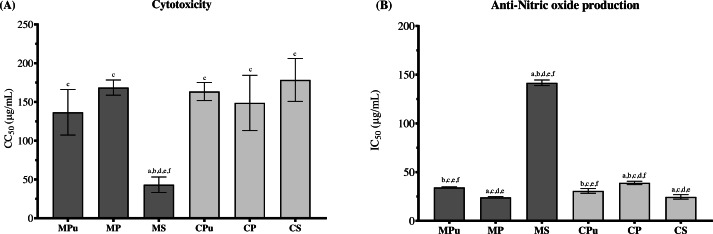
The cytotoxicity (A) of durian flour extracts represented by CC_50_ (50% cytotoxic concentration, while the anti-nitric oxide production activity (B) represented by IC_50_ (50% inhibitory concentration)) ^a^ Significantly different than MPu, ^b^ significantly different than MP, ^c^ significantly different than MS, ^d^ significantly different than CPu, ^e^ significantly different than CP, and ^f^ significantly different than CS.

Murine macrophage RAW 264.7 cells were used to determine the cytotoxicity of the durian flour extracts. Flour extract of Chanee seed (CS) had the lowest cytotoxicity followed by Monthong peel (MP), Chanee pulp (CPu), Chanee peel (CP), Monthong pulp (MPu) and Monthong seed (MS) with CC_50_ concentrations of 178.40 ± 27.68, 168.5 ± 9.82, 163.50 ± 11.71, 148.70 ± 35.66, 136.60 ± 29.28 and 43.23 ± 9.95 µg/mL, respectively (Fig. 3A). The cytotoxicity of MS was significantly different from MPu, MP, CPu, CP and CS, with *p* = 0.0064, 0.0001, 0.0002, 0.0078, and 0.0014, respectively. These results suggested that the flour extract of MS had the highest cytotoxicity on the macrophage cell line.

### Anti-inflammation of durian flour extracts

The anti-inflammatory activity of the durian flour extracts was tested by anti-nitric oxide (NO) production in lipopolysaccharide (LPS)-stimulated cells, and each durian flour extract was cotreated with LPS in murine RAW 264.7 macrophage cells. Flour extract of Monthong peel (MP) had the lowest IC_50_ value against NO production, indicating the highest anti-NO production activity among all tested flour samples (Fig. 3B). Its IC_50_ value was significantly different from those of Monthong pulp (MPu), Monthong seed (MS), Chanee pulp (CPu), and Chanee peel (CP), with *p* <0.0001, <0.0001, 0.0104 and 0.0002, respectively. However, it was not significantly different from Chanee seed (CS) (*p* = 0.75). The IC_50_ values sorted from low to high are: MP, CS, CPu, MPu, CP and MS (24.12 ± 0.78, 24.60 ± 2.29, 30.75 ± 2.40, 34.33  ± 0.58, 38.99 ± 1.75 and 141.70 ± 2.90 µg/mL, respectively).

## Discussion

Previous studies reported higher total polyphenols and flavonoids in Monthong than in Chanee when they were compared at the same ripening stage (Leontowicz et al., 2008; Toledo et al., 2008). The differences between the results in this study and in the previous studies are due to differences in fruit maturity stages, as this study used immature fruits and the previous studies used ripe fruits. No comparisons at the same ripening stage are available in the literature.

Ripe fruits had relatively higher concentrations of polyphenols and flavonoids than mature fruits and overripe fruits (Leontowicz et al., 2007). Moreover, our study found that the seeds and peels of durian fruits are potential sources of phenolic content. Therefore, the concept of processing unripe fruits and durian waste into useful products is reasonable and worth exploring for further development.

Fruits and vegetables are known as excellent sources of polyphenolic compounds. Polyphenols from durian extracts have a wide range of health benefits, including antioxidant effects (Chingsuwanrote et al., 2016). Our present study investigated their antioxidant capacities, including ABTS scavenging, nitric oxide (NO) scavenging, superoxide radical scavenging, hydroxyl radical scavenging and metal ion chelating. A lower SC_50_ value indicates a higher scavenging activity.

Phenolics are naturally occurring compounds in many plant species, including vegetables, cereals, fruits and traditional herbs. Previous studies found that phenolics were associated with antioxidant activity in many plant species. In medicinal plants, Phuyal and colleagues (2020) reported that extracts from the fruit, seed, and bark of *Zanthoxylum armatum* had the highest phenolic content, and their phenolic content was closely correlated with their antioxidant activity as determined by the DPPH method. Unfortunately, this previous study used only one method to test antioxidant activity. Several previous studies used different methods, such as the FRAP assay, DPPH assay, and ABTS assay (Aryal et al., 2019; Pokorná et al., 2015; Safriani et al., 2021). The results among the different studies showed a similar pattern. They were different in the magnitudes of the values for both phenolic content and antioxidant activity. In this study, five assays of antioxidant activity were used to test the different parts of durian.

Many previous studies have reported the antioxidant activity of various phytochemicals in durian. For example, Masturi and colleagues (2020) studied durian peels from local Indonesian durian (*Durio zibethinus*) and found terpenoids, phenolics and flavonoids, whereas alkaloids are not present in durian peels. Bambang and colleagues (2018) reported that ultrasonic-assisted extracts of durian peels contained total phenolics and total flavonoids. These authors also found strong correlations between these phytochemicals and antioxidant activity. Arancibia and colleagues (2008) studied the compositions of phytochemicals in durian at different maturity stages, including mature, ripe, and overripe, using high-performance liquid chromatography with diode array detection (HPLC/DAD). Campherol was the highest at all maturity stages, whereas vanillic acid, cinnamic acid, morin and myricetin were found at all maturity stages. Quercetin was detected at the ripe stage, and p-coumaric acid was not detected at the overripe stage, but it was detected at other maturity stages. Apigenin and caffeic acid were not detected at any maturity stage. The results of a previous study indicated that polyphenols, phenolic acids, flavonoids and volatile acids are the major phytochemicals playing an important role in antioxidant activity in durian (Arancibia et al., 2008).

The antioxidant mechanisms of phenolics involve many possible mechanisms. For example, reacting with a variety of free radicals and donating hydrogen from hydroxyl groups will react with oxygen and reactive nitrogen species, resulting in disruptions of new radical generation. Moreover, phenolic compounds can transfer a single electron, and sequential proton loss electron transfer and chelated metal ions are involved in the production of free radicals (Pereira et al., 2009; Zeb, 2020). According to the various antioxidant mechanisms, several antioxidant assays were performed in our present study.

The results indicated that flour extracts from pulp, peel, and seeds of the two durian varieties used in this study had antioxidant capacities. Although the antioxidant activities determined by the different methods were different, the results clearly confirmed that antioxidant activities were present in parts of the fruits in addition to the pulp, which was already reported in previous studies. Therefore, the inner peel and seed are also useful for the production of other products with health benefits. In a review in 2019, the authors collected information regarding the bioactive compounds and antioxidant capacities of many durian cultivars in Thailand and other countries in Southeast Asia. Previous studies also reported the variations among durian varieties, including Monthong and Chanee, in antioxidant capacities determined by different methods (Aziz & Mhd Jalil, 2019). Our results supported a previous report regarding antioxidant activity in durian varieties in Southeast Asia, especially Monthong and Chanee, which were also used in our current study. The results of this study will provide novel knowledge to the durian research community regarding the presence of antioxidant activity in the inner peel and seeds of durian, and this can lead to more research on reducing waste during durian production.

The present study indicated that flour extracts from durian had potent NO inhibitory activity. It also demonstrated that phenolic compounds might be partially responsible for the NO inhibitory activity of durian. Flour extracts of Chanee had lower cytotoxicity and higher potent NO inhibitory activity than those of Monthong. The results in this study agree with those in a previous study. Feng and colleagues reported that triterpenoids and glycosides from durian peel exerted inhibitory activities on lipopolysaccharide-induced NO production in RAW 264.7 cells (Feng et al., 2018). According to Moore and colleagues in cranberry, polyphenols and volatile extracts inhibited the evolution of nitric oxide in RAW 264.7 cells, which were activated by lipopolysaccharide (LPS) (Moore et al., 2019).

Similarly, Chingsuwanrote and colleagues reported the anti-inflammatory activity of the ripe pulp of Monthong and Chanee. Researchers have shown that Monthong extracts are more effective than Chanee extracts in reducing ROS production and decreasing the secretion of tumor necrosis factor alpha (TNF-α) and interleukin-8 (IL-8). Monthong extract was a more effective anti-inflammatory than Chanee extract (Chingsuwanrote et al., 2016). These results were in agreement with those in other plant species. In the fruits of *Cornus officinalis* (Cornaceae), phenolic compounds could inhibit the development of nitric oxide in RAW 264.7 macrophage cells, which were activated by LPS (Thu et al., 2021). Furthermore, these compounds showed appreciable anti-inflammatory activity.

The antioxidant activities of durian flour extracts in our present study indicated that Monthong seed (MS) was more effective than the extracts from other parts. It had the best activity for scavenging nitric oxide and hydroxyl radicals. However, it was not as good at scavenging superoxide and chelate metal ions, and was particularly not good at scavenging ABTS radicals. Its antioxidant activities were not consistent with its cytotoxicity and anti-NO production abilities. MS had the highest cytotoxicity and significantly lower anti-NO production than any of the other extracts from durian flour.

This research provides basic information on the antioxidant and anti-inflammation properties of the pulp, peel and seed of durian, and the information provided is useful for the durian industry. However, further investigations into the applications of durian flours as a food ingredient are still required because the ability of the flour extracts to replace conventional flours is still unknown and the different flour extracts have different properties.

## Conclusions

The extracts of six flour samples made from unripe pulp, inner peel, and seeds of two durian varieties, Monthong and Chanee, had high total phenolic contents (TPCs) in the range of 3180.32 to 5285.37 mg GAE/g. Chanee pulp (CPu) had the highest TPC of 5285.37 ± 517.65 mg GAE/g. However, the flour samples made from Monthong, including Monthong pulp (MPu), Monthong peel (MP) and Monthong seed (MS), had better antioxidant capacities than that of Chanee. The extract of Chanee seed (CS) had the lowest cytotoxicity against mouse macrophage RAW.264.7 cells. Moreover, the extract of MP also had the highest anti-inflammatory activity and the lowest IC_50_ against LPS-induced nitric oxide (NO) production by macrophage RAW 264.7 cells. These results indicate that the flour derived from all parts of the two durian varieties had antioxidant and anti-inflammatory activities.

## Supplemental Information

10.7717/peerj.12933/supp-1 Supplemental Information 1 Raw data

## Additional Information and Declarations

### Data Availability

The following information was supplied regarding data availability: The raw measurements are available in the Supplementary File.
